# Foliar selenium fertilization alters the content of dietary phytochemicals in two rocket species

**DOI:** 10.3389/fpls.2022.987935

**Published:** 2022-08-31

**Authors:** Michela Schiavon, Serenella Nardi, Elizabeth A. H. Pilon-Smits, Stefano Dall’Acqua

**Affiliations:** ^1^Department of Agricultural, Forest and Food Sciences (DISAFA), University of Turin, Grugliasco, TO, Italy; ^2^Department of Agronomy, Food, Natural Resources, Animals and Environment (DAFNAE), University of Padova, Legnaro, PD, Italy; ^3^Department of Biology, Colorado State University, Fort Collins, CO, United States; ^4^Department of Pharmaceutical and Pharmacological Sciences, University of Padova, Padova, Italy

**Keywords:** rocket, biofortification, selenate, sulfur, glutathione, glucosinolates, amino acids, phenolics

## Abstract

Biofortification is the process that aims to enrich crops in micronutrients and valuable compounds. Selenium (Se) biofortification has particularly attracted increasing interest in recent times due to the growing number of individuals suffering from Se deficiency. Selenate and selenite are the Se forms most frequently administered to crops. In this study, Se was applied foliarly as selenate at 2.5, 5, or 10 mg per plant to two rocket species, *Diplotaxis tenuifolia* and *Eruca sativa*, grown in soil and the effects in terms of Se enrichment and content of primary and secondary metabolites were comparatively analyzed. We also compared our results with those obtained previously when selenate was supplied to the same species in hydroponics by addition to the nutrient solution. In most cases, the results were the opposite. In *E. sativa*, foliar Se treatment was more effective in promoting Se accumulation, sulfur (S), cysteine, and glucosinolates. No significant effect of Se was evident on total phenolic content, but there were individual phenols. Among amino acids, the content of proline was increased by Se, perhaps to counteract osmotic stress due to high Se accumulation. In *D. tenuifolia*, the content of S and cysteine decreased under Se treatment, but the amount of glutathione was steady, suggesting a preferred assimilation of cysteine toward the synthesis of this antioxidant. Consistent, the content of methionine and glucosinolates was reduced. The content of total phenolics was enhanced only by the low Se dosage. In both species, selenocysteine (SeCys) was identified, the content of which was higher compared to plants grown hydroponically. Concluding, most metabolic differences between rocket species were observed at high Se supplementation. Low Se foliar fertilization was effective in an enriching rocket in Se without affecting other phytochemicals. However, the Se dosages sufficient for biofortification could be even lower, as the Se concentration in rocket treated with 2.5 mg Se per plant was still very high and the edible part should not be eaten undiluted. Also, a single method of Se supplementation does not appear to be optimal for all plant species or the same species, as the metabolic responses could be very different.

## Introduction

Biofortification is the process of adding vital nutrients and health-promoting compounds to crops to improve their nutritional value and enrich the diet of vulnerable populations who frequently have a plant-based diet (White and Broadley, 2009; Zhao and McGrath, 2009; Wu et al., 2015). An emerging area of research focuses on strategies that aim to increase the content of selenium (Se) in staple crops and other vegetables containing low amounts of this element in their edible parts (Schiavon et al., 2020). An estimated 1 billion people have a sub-optimal Se intake in the diet (Combs, 2001), and this number is expected to increase in the future due to the impact of climate change on agriculture (Jones et al., 2017). The resulting Se-biofortified crops can additionally be enriched in other phytochemicals, such as minerals and antioxidant constituents, creating high value vegetables that offer a variety of benefits to consumers (Newman et al., 2019; D’Amato et al., 2020; Schiavon et al., 2020).

Selenium is an essential micronutrient for humans, and the recommended intake is of 55–70 μg per day (World Health Organization, 2009; USDA–ARS, 2012); it is also essential for several animals and microorganisms (Kieliszek, 2019), while its role is different for plants being non-essential (Schiavon and Pilon-Smits, 2017). Plants uptake Se from soil and can transform the inorganic Se into the organic forms, namely the amino acids selenocysteine (SeCys) and selenomethionine (SeMet), but do not possess specific mechanisms for their further insertion in selenoproteins with critical roles in metabolism (White, 2016, 2018). Rather, Se-amino acids are misincorporated in proteins in place of their sulfur (S) analogs cysteine (Cys) and methionine (Met), thus causing protein misfolding and loss of function (Sabbagh and Van Hoewyk, 2012; Van Hoewyk, 2013). In addition, Se compounds at high concentration prompt oxidative stress in cells due to reactive oxygen species (ROS) overgeneration and disruption of reactive nitrogen species (RNS) that leads to protein tyrosine nitration (Kolbert et al., 2016; Gupta and Gupta, 2017). On the other hand, Se at low concentration is recognized as beneficial for many plants by stimulating their growth and antioxidant systems (Chauhan et al., 2019).

Different agronomic and genetic biofortification approaches can be used to increase Se concentration in crops and their success relies on multiple factors, such as the form and dosage of Se applied, the mode of Se administration, the crop species and variety, and the plant growth system (soil or hydroponics) (Ros et al., 2016; Bañuelos et al., 2017). Plants can absorb different forms of Se, either inorganic (e.g., selenate, selenite) or organic (e.g., Se-amino acids). However, inorganic Se salts are more commonly employed in biofortification programs (Schiavon et al., 2020), but they must be applied in small quantities, and for this reason they are often added to fertilizers that act as carriers of Se (Ramkissoon et al., 2019). Selenium fertilizers can be applied to soil to increase the amount of Se available to plants; this method is relatively inefficient: only 12% of soil-applied Se fertilizers were absorbed via root uptake in a study by Broadley et al. (2010). Alternatively, foliar Se administration to plants grown in soil or the addition of Se to the nutrient solution within hydroponic systems could be exploited for biofortification; both methods offer the advantages of fast Se uptake and assimilation by plants, and avoid immobilization processes of Se compounds that may happen in soil. The application of Se via foliar spray also prevents the need of Se root-to-shoot translocation to the edible aboveground organs (Ros et al., 2016; Ramkissoon et al., 2019). The use of Se-laden material derived from Se hyperaccumulator plants as a green manure or growing crops in Se-rich soils are other, but still very limited, options (Bañuelos et al., 2015, 2017; Schiavon et al., 2020).

Plants with the potential to accumulate appreciable amounts of Se in their edible parts are regarded as potential candidates for successful biofortification. Crops belonging to the Brassicaceae are interesting in this respect: they are defined as secondary Se accumulators, based on their capacity to accumulate and tolerate up to 1,000 μg Se g^–1^ d.wt. (White, 2018). These plants have high S content in their tissues, which besides essential S compounds included secondary S compounds, such as glucosinolates (GLS), which play defensive roles against herbivores (Agerbirk and Olsen, 2012; Jørgensen et al., 2015). These compounds also exert protective roles in humans (Melrose, 2019). Owing to the chemical similarity of S to Se, the administration of Se to plants could result in a decrease in S content and, in turn, depletion of primary and secondary S compounds (Robbins et al., 2005; Schiavon et al., 2013; Bachiega et al., 2016). However, sometimes Se may also stimulate the S uptake and assimilation pathway, leading to higher S levels. In this respect, contrasting results have been reported, even within the same plant species, when using different experimental setups for biofortification (Schiavon et al., 2016).

Previously, we evaluated the effect of Se on the capacity of two rocket species (perennial wall rocket, *Diplotaxis tenuifolia* (L.) DC., and annual garden rocket, *Eruca sativa* Mill.) Grown in hydroponics to accumulate Se depending on interactions with S uptake and assimilation (Dall’Acqua et al., 2019). We also assayed the effect of Se addition to the nutrient solution on the synthesis and accumulation of GLS, phenolic compounds and amino acids. In this study, we aimed to evaluate the effect of different selenate dosages applied via foliar spray on the same rocket species, but cultivated in soil pots, to investigate potential differences in Se accumulation and metabolic-related outcomes depending on the type of Se administration. Indeed, despite the cultivation of plants in hydroponics offers several advantages (e.g., water saved, growth controlled over climate changes, optimal use of nutrients, reduced pests and diseases, and absence of competition with weeds), it is very expensive to manage and requires investment, thus it is not affordable in poor countries where biofortification programs should be more extensively conducted.

We again used *E. sativa* and *D. tenuifolia* for consistency, and because these species differ in the content of health-promoting phytochemicals, have a wide distribution, and are of increasing importance after the circulation of the ready-to-use salads in the vegetable retail markets (Heimler et al., 2007; Caruso et al., 2020). The species *E. sativa*, in particular, has been artificially selected and this may have led to some biochemical and physiological differences with *D. tenuifolia*.

## Materials and methods

### Experimental setup

Seeds of *E. sativa* and *D. tenuifolia* (Corona sementi, Mortegliano, UD, Italy) were sown in 1 L-pots placed inside a greenhouse under natural light conditions (April to May, average day/night temperature 18/15°C and photoperiod 14/10 h). The pots were filled with peat, soil, and perlite in the ratio 60:30:10, watered twice a day and each contained a germinated plant. Once plants were 6 weeks old, they were divided in four groups (10 pots per group) containing 10 plants each. Three of these groups received a unique foliar application of selenium in the form of sodium selenate (Na_2_SeO_4_) at dosages of 2.5, 5, or 10 mg per plant. Se-containing solutions differing in selenate concentration (250, 500, and 1,000 mg/L) were prepared and sprayed on the leaves in order to apply them at the same volume (10 ml) to each plant. Plants were quite similar in leaf size, but to avoid dripping from the leaves we applied the selenate solution in two times (5 ml each time) at 1 h interval. One group of plants was sprayed with an equal volume of water and used as a control. During foliar Se treatment, the soil surrounding the plants was covered to avoid any contamination with Se. Plants were harvested 10 days after the Se treatment was applied, washed with distilled water and dried with blotting paper. Specifically, leaves from each plant were immersed in water for about 5 min and then rinsed two times under running distilled water. For roots, at least 5 min were first required to gently clean them from the soil particles. Then, they were subjected to the same procedure as leaves. Six plants per treatment were divided into roots and shoots and their fresh weight was measured individually. The plant material was then placed inside a drying oven for 2 days at 70°C to measure the dry weight. The leaves and roots of the remaining plants were frozen in liquid nitrogen and kept at –80°C for further analyses. The experimental design for plant growth was randomized (the pots were re-arranged three times a week) and the entire experiment was replicated two times. Data were not pooled together from the two experiments to get means, but the trend of Se, S, and metabolic compounds was confirmed by the second experiment.

### Determination of total selenium, carbon, nitrogen, and sulfur in soil

Samples of soil dried at room temperature were analyzed for carbon, nitrogen, and sulfur contents using an elemental analyzer (Vario MACRO CNS, Hanau, Germany). For total Se determination, dried soil samples were extracted with HNO_3_/HCl (ratio 1:3v/v) and warmed until boiling for 30 min under agitation. Samples were then filtered (0.45 μm, Millipore), and the quantification of Se was performed via inductively coupled plasma atomic emission spectroscopy (ICP-AES) (Fassel, 1978). Analyses were conducted in triplicates.

### Determination of total selenium and sulfur in plants

Leaf and root tissues of rocket plants were dried for 48 h at 80°C and further digested using nitric acid according to the method reported by Zarcinas et al. (1987). Inductively coupled plasma atomic emission spectroscopy was used according to the protocol by Fassel (1978) to determine each digest’s Se and S elemental concentrations, using appropriate standards and quality controls. Analyses were conducted in triplicates (1 replicate = 1 plant).

### Identification and quantification of glucosinolates

Glucosinolates were extracted from rocket leaves according to the protocol reported by Schiavon et al. (2016). To prevent myrosinase activity in the samples, glucosinolates were extracted from 6 g of leaves boiled for 4 min in 18 ml of a methanol/water solution (ratio 70:30, v/v). Sinigrin (1.26 mg/ml) was added as internal standard to this solution. To achieve the complete glucosinolates extraction, leaf material residual after sample filtration was re-extracted using 70% (v/v) methanol for 4 min. The two extracts from each sample were further combined and purified through a Solid-Phase Extraction (SPE) column (0.8 cm × 4 cm, Agilent Technologies) equipped with 0.256 g of an ion-exchange resin (DEAE-SEPHADEX-A25) imbibed in 4 ml of a 0.5 M Na-acetate buffer solution (pH = 5). The column was washed with 1 ml deionized H_2_O and then loaded with 2.5 ml extract containing the internal standard (Sinigrin). The further purification steps were performed according to the protocol reported by Schiavon et al. (2016).

The analysis of glucosinolates was performed in High Performance Liquid Chromatography - Mass Spectrometry (HPLC-MS) on a Varian LCMS 500 Ion Trap equipped with Electrospray Ionization (ESI) as a source operating in positive ion-mode. The analysis of the fragmentation patterns of spectra shown in Supplementary Table 1 was realized through the Turbo Detection Data Scanning (TDDS) function. The chromatographic separation was performed in an Agilent 1,260 Liquid Chromatography (LC) system using a column Eclipse XDB C-8 5 μm 2.1 mm × 150 mm as described by Schiavon et al. (2016). For the quantification of glucosinolates, glucoerucin was used as a reference standard at different concentration levels. Analyses were performed on three biological replicates (1 replicate = 1 plant).

### Determination of low molecular weight thiol compounds

Frozen leaf material (250 mg) was ground in liquid nitrogen with 0.1 N HCl and 1 mM Ethylenediaminetetraacetic acid (EDTA). Extracts were centrifuged at 10,000 g for 10 min and then analyzed for low-molecular-weight (LMW) thiol contents. Extracts (50 μL) were further derivatized using 7-Fluorobenzofurazan-4-sulfonic acid ammonium salt (SBD-F) fluorophore (Sigma-Aldrich, St. Louis, MO, United States). Low-molecular-weight thiols (cysteine and total glutathione) were separated by isocratic HPLC according to Masi et al. (2002). The mobile phase was 3% methanol in 75 mM NH_4_^+^ formiate, pH 2.9. Analyses were performed on three biological replicates (1 replicate = 1 plant).

### Quantification and identification of free amino acids

Free amino acids were determined in rocket leaves instead of total amino acids to get preferential information about metabolism rather than the function of gene expression. Extraction of free amino acids, including Se-amino acids, was obtained from three replicates of frozen rocket leaves (500 mg) using 0.1 M HCl (1:4, w/v). The extracts underwent centrifugation at 4°C for 10 min at 10,000 g. The supernatants were collected and filtered at 0.45 μm (Millipore). Qualitative and quantitative analyses of amino acids were realized through HPLC-MS using a Varian Liquid Chromatography - Mass Spectrometry (LC-MS) 500 equipped with a ZORBAX Eclipse Plus AAA column (3.5 μm × 3 mm × 150 mm) as described by Schiavon et al. (2016). The identification and quantification of the amino acids in the extracts were attained via Ion Trap Mass Spectrometry (Varian 500 MS) coupled to the HPLC system, by comparison with appropriate standards and analysis of the fragmentation patterns of spectra (data not shown) through the TDDS function. For the identification and quantification of the amino acids, the reference standards consisted of these amino acids: Alanine, Arginine, Asparagine, Glutamine, Glutamic acid, Glycine, Histidine, Isoleucine, Leucine, Lysine, Methionine, Phenylalanine, Proline, Serine, Threonine, Tryptophan, Tyrosine, Valine, Selenomethionine, Selenocysteine, and Se-Methyl-Selenocysteine. The free amino acids whose content was below the detection limit are not reported in Table 1. Analyses were performed on three biological replicates (1 replicate = 1 plant).

**TABLE 1:** Effects of selenate treatment on the content of selected amino acids in leaves of rocket species (*Eruca sativa* and *Diplotaxis tenuifolia*) grown in soil and treated foliarly with selenate dosages ranging from 0 to 10 mg per plant.

Amino acid (mg/100 g FW)	0	2.5	5	10
***Eruca sativa* Se treatment (mg per plant)**				
Phenylalanine	1.50 ± 0.10b	1.36 ± 0.19ab	1.64 ± 0.14ab	1.84 ± 0.16a
Isoleucine	0.45 ± 0.02b	0.82 ± 0.14a	0.18 ± 0.05c	0.13 ± 0.02c
Leucine	0.42 ± 0.03b	0.65 ± 0.09a	0.20 ± 0.12c	0.08 ± 0.02c
Histidine	2.30 ± 0.39b	3.83 ± 0.50a	4.00 ± 0.37a	4.40 ± 0.28a
Tyrosine	0.14 ± 0.01a	0.18 ± 0.04a	0.15 ± 0.05a	0.18 ± 0.05a
Tryptophan	0.35 ± 0.10a	0.40 ± 0.13a	0.30 ± 0.15a	0.32 ± 0.10a
Arginine	1.53 ± 0.22d	0.85 ± 0.06c	0.45 ± 0.02b	0.27 ± 0.07a
Alanine	5.83 ± 1.84bc	3.77 ± 0.77c	5.64 ± 0.66b	11.46 ± 3.36a
Valine	2.32 ± 0.68b	3.74 ± 0.52a	3.96 ± 0.31a	3.08 ± 0.15ab
Lisine	1.18 ± 0.05a	1.11 ± 0.08a	1.06 ± 0.07a	1.20 ± 0.07a
Proline	18.07 ± 3.44b	34.06 ± 8.61a	21.94 ± 7.17ab	20.55 ± 5.80ab
Methionine	0.15 ± 0.03a	0.11 ± 0.05a	0.14 ± 0.04a	0.17 ± 0.04a
Se-cysteine	0.00 ± 0.00c	5.86 ± 0.64a	5.58 ± 0.13a	4.23 ± 0.51b
***Diplotaxis tenuifolia* Se treatment (mg per plant)**				
Phenylalanine	1.19 ± 0.05b	1.19 ± 0.05b	1.19 ± 0.05b	1.19 ± 0.05b
Isoleucine	0.09 ± 0.02c	0.18 ± 0.01b	0.13 ± 0.02c	0.36 ± 0.08a
Leucine	0.09 ± 0.01b	0.13 ± 0.03 ab	0.11 ± 0.01b	0.20 ± 0.03a
Histidine	3.70 ± 0.26a	4.00 ± 0.39a	3.73 ± 0.13a	4.58 ± 0.19a
Tyrosine	0.17 ± 0.01a	0.17 ± 0.01a	0.17 ± 0.01a	0.17 ± 0.01a
Tryptophan	0.31 ± 0.12a	0.31 ± 0.12a	0.31 ± 0.12a	0.31 ± 0.12a
Arginine	1.20 ± 0.14a	1.33 ± 0.16a	0.95 ± 0.12a	1.17 ± 0.27a
Alanine	6.31 ± 0.67a	6.62 ± 1.14ab	3.66 ± 0.10c	5.03 ± 0.81b
Valine	2.75 ± 0.91a	2.46 ± 0.50a	2.40 ± 0.32a	3.15 ± 0.96a
Lisine	1.20 ± 0.06a	1.17 ± 0.06a	1.08 ± 0.02a	1.10 ± 0.05a
Proline	20.76 ± 6.05a	21.30 ± 1.80a	14.25 ± 3.08ab	13.81 ± 1.71b
Methionine	0.24 ± 0.04a	0.21 ± 0.04a	0.25 ± 0.03a	0.06 ± 0.02b
Se-cysteine	–	4.89 ± 0.74a	4.89 ± 0.74a	4.89 ± 0.74a

Different letters along rows indicate significant differences (*p* < 0.05, ±STD) among treatments.

### Identification and quantification of polyphenols

Extraction of polyphenols from three replicates of frozen rocket tissues was performed using methanol: water (1:1, v/v) solution in ultrasonic bath for 15 min. The ratio of plant material to the mixture was 1:10 (w/v) and extracts were filtered at 0.45 μm (Millipore). Validation of the extraction procedure was realized by measuring the recovery percentage of chlorogenic acid and rutin in replicates of leaf samples.

Qualitative and quantitative analyses of polyphenols were realized both via HPLC-MS and High Liquid Chromatography with Diode Array detector (HPLC-DAD). For the separation of polyphenols, an Eclipse Plus C-18 column (3.5 μm × 2.1 mm × 150 mm, Agilent) was used in the HPLC system Varian 212 at 35°C as reported in Schiavon et al. (2016). The identification and quantification of the principal polyphenols in the extracts were conducted via Ion Trap Mass Spectrometry (Varian 500 MS) coupled to the HPLC system, by comparison with appropriate standards (chlorogenic acid for phenols, rutin for flavonoids) and analysis of the fragmentation patterns of spectra (Supplementary Table 2) through the TDDS function. Electrospray Ionization was used as a source in negative ion-mode and the mass range considered was within 50–3,500 uma. Each sample’s volume injected was equal to 10 μL. Analyses were performed on three biological replicates (1 replicate = 1 plant).

### Statistical analysis

Analysis of variance (ANOVA) was performed using the SPSS software, and was followed by pair-wise *post-hoc* analyses (Student–Newman–Keuls test) to determine which means differed significantly at *p* < 0.05 (+STD).

## Results

### Plant growth in response to selenium application

**FIGURE 1:**
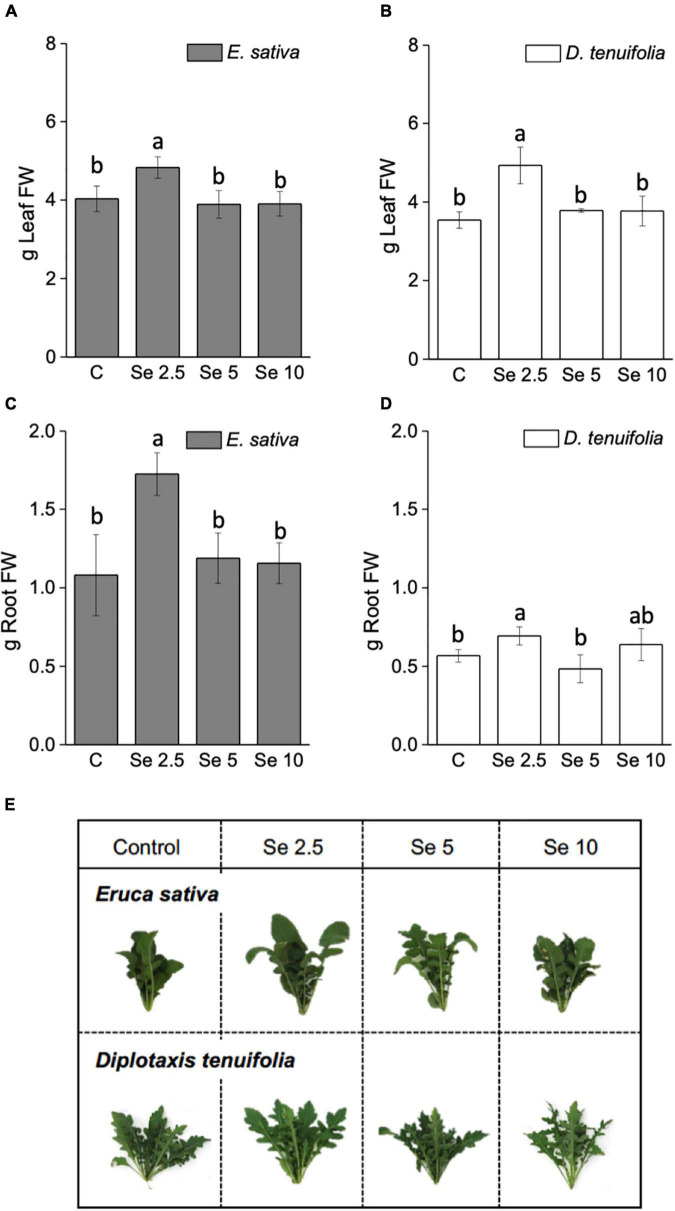
Fresh weight (FW) of leaves **(A,B)** and roots **(C,D)** of *E. sativa* and *D. tenuifolia* plants grown in soil and subjected to foliar fertilization with selenate dosages ranging from 0 to 10 mg per plant. The FW reported is the average FW of each leaf (±SD, *n* = 6). Different letters above bars indicate significant differences between the means (*p* < 0.05). **(C,E)** Images of representative rocket species fertilized with selenate.

Foliar application of Se at the minimum dosage (2.5 mg Se per plant) increased the fresh leaf and root biomass of both rocket species, while no significant effect on growth was observed when higher Se doses (5 and 10 mg Se per plant) were administered to the plants (Figures 1A,B). The increment in leaf biomass was more pronounced for *D. tenuifolia* (+28.2 vs. 16.6.%), while the root biomass was more enhanced in *E. sativa* (+37.5 vs. 14.9%) (Figures 1C,D). A similar trend was evident for plant dry weight (data not shown). Figure 1E depicts representative rocket plants fertilized with different amounts of Se.

### Selenium accumulation and effects on sulfur and thiol compounds

**FIGURE 2:**
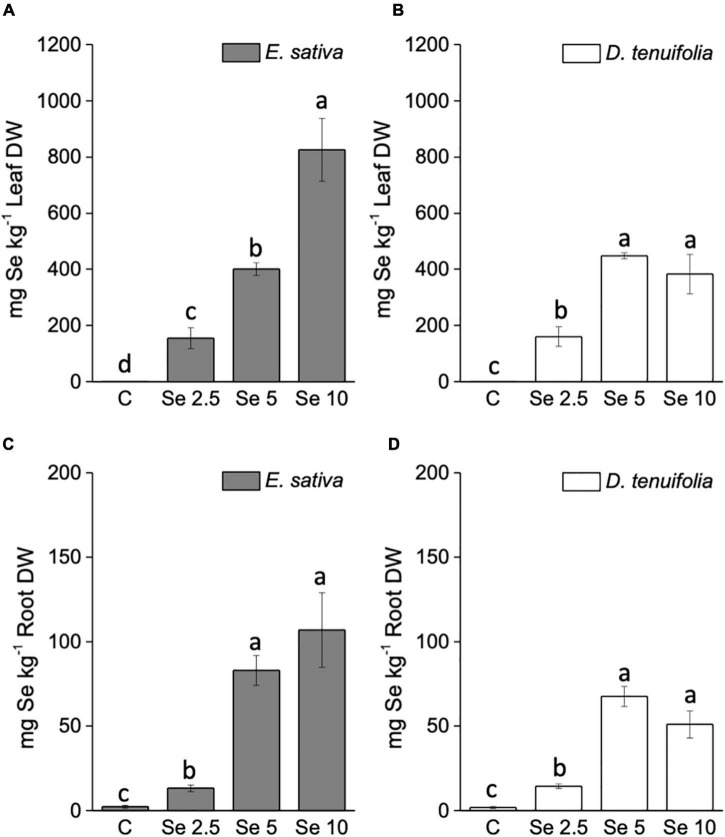
Selenium concentration in leaves **(A,B)** and roots **(C,D)** of *E. sativa* and *D. tenuifolia* plants grown in soil and subjected to foliar fertilization with selenate dosages ranging from 0 to 10 mg per plant. Data shown are the mean ± SD of three replicates. Different letters above bars indicate significant differences between the means (*p* < 0.05).

The concentration of Se in leaves and roots of *E. sativa* positively correlated with the amount of applied Se (Figures 2A,B), while in *D. tenuifolia* this type of correlation was determined only when plants received up to 5 mg Se per plant (Figures 2C,D). The application of a higher Se dosage (10 mg per plant) did not further increase the Se content in *D. tenuifolia*, as a plateau was distinctly achieved. The two species contained similar Se concentrations when supplied with 2.5 or 5 mg Se per plant. *E. sativa* accumulated about 2-fold more Se at a higher Se dosage compared to *D. tenuifolia*. In general, plants accumulated approximately 7.5 times more Se in leaves than roots.

**FIGURE 3:**
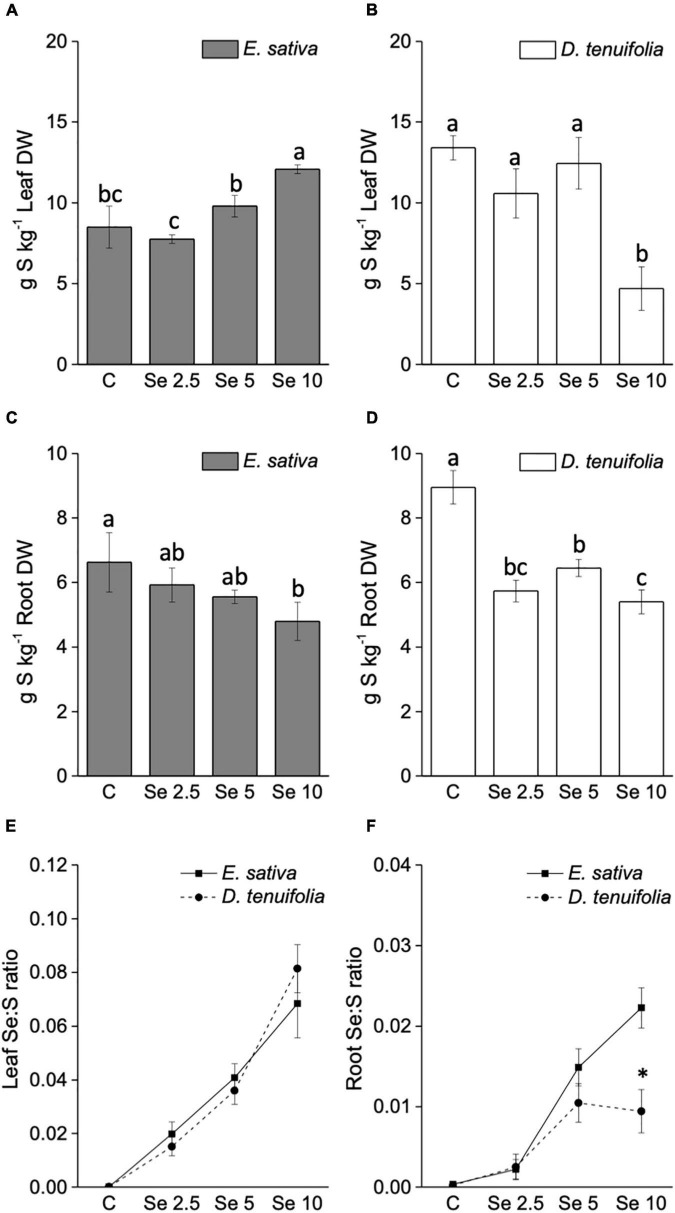
Sulfur concentration in leaves **(A,B)** and roots **(C,D)** of *E. sativa* and *D. tenuifolia* plants grown in soil and subjected to foliar fertilization with selenate dosages ranging from 0 to 10 mg per plant. Se:S ratio of leaves **(E)** and roots **(F)** of the two rocket species. Data shown are the mean ± SD of three replicates. Different letters above bars indicate significant differences between the means (*p* < 0.05). In the line graph, the asterisks indicate significant differences in the root Se:S ratio between the two rocket species.

The trend of S content in response to Se application was contrasting between the two species (Figures 3A,B). With respect to *E. sativa*, the S leaf content increased with the increase of applied Se, while a decline was evident in the roots. In contrast, the Se administration to *D. tenuifolia* caused a significant depletion of S. This effect was determined in leaves fertilized with 10 mg Se per plant, and in roots at any Se dosage applied (Figures 3C,D).

The Se:S ratio was overall higher in leaves than in roots (Figures 3E,F). The leaf Se:S ratio was comparable between *E. sativa* and *D. tenuifolia* at any Se dosage applied, but the values in roots were greater for *E. sativa* plants supplemented with 10 mg Se per plant. This is because, although S decreased in both species, *E. sativa* contained more Se in roots than *D. tenuifolia*.

**FIGURE 4:**
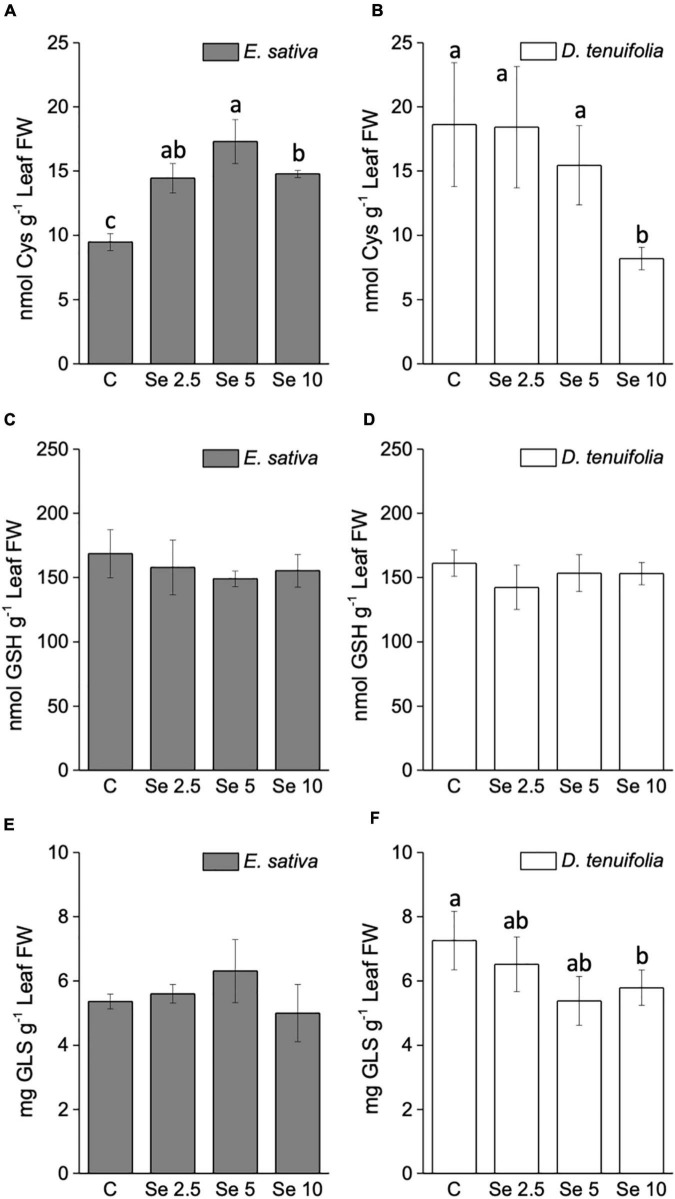
Content of cysteine **(A,B)**, total glutathione **(C,D)** and total glucosinolates **(E,F)** in leaves of *E. sativa* and *D. tenuifolia* plants grown in soil and subjected to foliar fertilization with selenate dosages ranging from 0 to 10 mg per plant. Data shown are the mean ± SD of three replicates. Different letters above bars indicate significant differences between the means (*p* < 0.05).

In line with the trend of S accumulation, the leaf content of the amino acid cysteine (Cys) increased in *E. sativa* plants sprayed with Se (Figure 4A), whereas it was depleted in D. *tenuifolia* (Figure 4B). The leaf content of glutathione (GSH) was almost unchanged by Se fertilization in both rocket species (Figures 4C,D).

### Effects of selenium application on the amount of total and individual glucosinolates

The total glucosinolate (GLS) content did not vary in response to low (2.5 mg Se per plant) Se application in either of the rocket species (Figures 4E,F). However, the supplementation of higher Se dosages led to an increase of GLS in *E. sativa*, and a decrease in *D. tenuifolia*.

**FIGURE 5:**
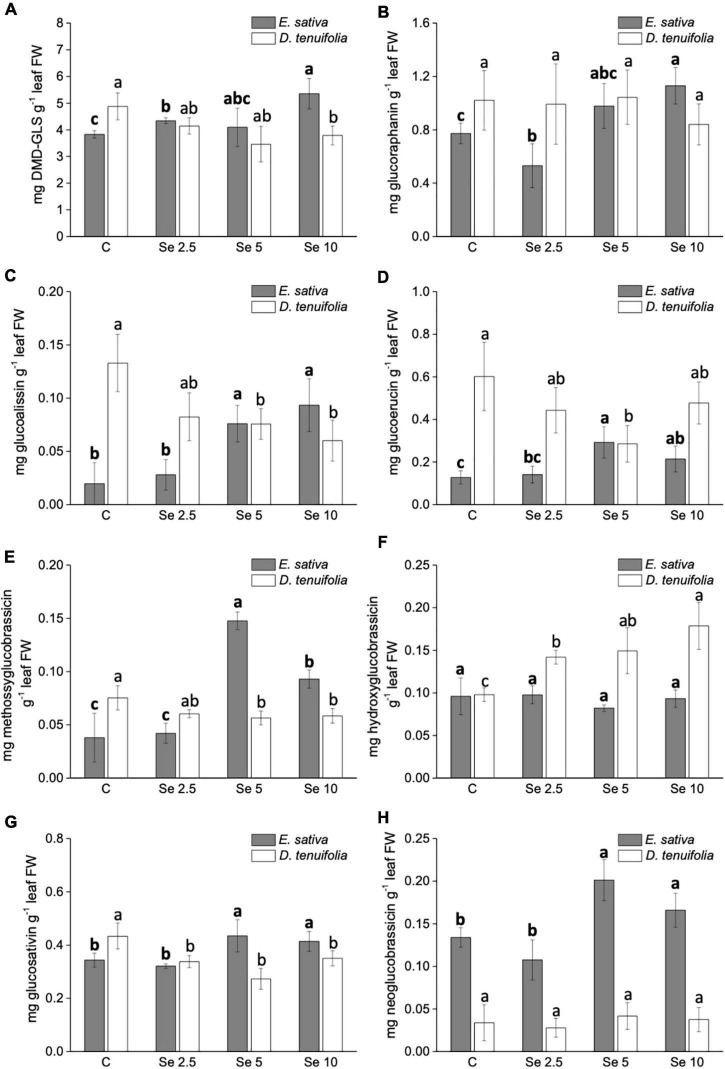
Effects of selenate treatment on the content of individual glucosinolates, i.e., DMD-GLS **(A)**, glucoraphanin **(B)**, glucoalissin **(C)**, glucoerucin **(D)**, methossiglucobrassicin **(E)**, hydroxyglucobrassicin **(F)**, glucosativin **(G)**, and neoglucobrassicin **(H)**, identified in *Eruca sativa* and *Diplotaxis tenuifolia* plants grown in soil and subjected to foliar fertilization with selenate dosages ranging from 0 to 10 mg per plant. Data shown are the mean ± SD of three replicates. Different letters in bold above bars indicate significant differences between the means (*p* < 0.05) of values referred to *E. sativa*, while different letters not bolded indicate significant differences between the means (*p* < 0.05) of values referred to *D. tenuifolia*. DBM-GLS = Dimeric-4-mercaptobutyl glucosinolate.

The GLS identified in both species are listed in Supplementary Table 1 and mainly consisted of aliphatic GLS derived from methionine. We did not detect any Se-GLS, though they have been identified in other species (McKenzie et al., 2019). The most abundant was DMD (Dimeric-4-mercaptobutyl)-GLS, followed by glucoraphanin, glucosativin and glucoerucin (Figures 5A–D). In *E. sativa*, the content of most GLS (glucoerucin, glucoraphanin, DMD-GLS, glucoalissin, methoxy glucobrassicin, glucosativin, and neoglucobrassicin) was enhanced by Se fertilization, especially when Se was supplemented to plants at 5 or 10 mg per plant (Figures 5A–F). Neoglucobrassicin was more abundant in *E. sativa* than *D. tenuifolia* (Figure 5G), and the content of hydroxyglucobrassicin did not vary in response to Se administration (Figure 5H). In *D. tenuifolia*, however, the glucoerucin content was higher than in *E. sativa*, but the application of 5 or 10 mg Se per plant substantially decreased it (Figure 5D). A similar decreasing trend was observed in *D. tenuifolia* for DMD-GLS, glucosativin, glucoalissin, and methoxy glucobrassicin (Figures 5A,C,E,F), while the glucoraphanin and neoglucobrassicin contents were almost unchanged (Figures 5B,G). The only GLS of *D. tenuifolia* whose content increased after Se supplementation was hydroxyglucobrassicin (Figure 5H).

### Effects of selenium application on the content of free amino acids

The foliar fertilization with Se determined different effects on the abundance of single amino acids in both species (Table 1). In *E. sativa*, the content of leucine, isoleucine and proline was increased by the low Se dosage (2.5 mg Se per plant). Leucine and isoleucine were then reduced by higher Se dosages compared to the Se untreated plants. Histidine and valine were more accumulated with any dosage of Se applied, while phenylalanine and alanine were only by 10 mg Se per plant. In contrast, the amount of arginine was depleted by Se fertilization, while no effect was evident on tyrosine, tryptophan and lysine. In *D. tenuifolia*, the content of leucine, isoleucine and histidine increased with increasing Se dosages. Proline, alanine and lysine were decreased in plants sprayed with 5 or 10 mg Se per plant. The amount of valine and arginine and of the aromatic amino acids tyrosine and tryptophan was not affected by Se, but phenylalanine was more abundant in plants fertilized with 10 mg Se per plant.

Among Se amino acids, only SeCys was detected and its content was comparable in *E. sativa* and *D. tenuifolia*. Notably, the SeCys content declined in both species with high Se supplementation.

### Effects of selenium application on the amount of single and total polyphenolic compounds

Variation in the leaf content of single polyphenols identified in *E. sativa* and *D. tenuifolia* is reported in Table 2. These compounds consisted mainly of flavonoid derivatives. Specifically, glycosylated derivatives of kaempferol and isorhamnetin, often esterified with phenylpropanoid acids, were dominant in *E. sativa*, especially kaempferol-3,4′-diglucoside and kaempferol-3-sin a poil triglucoside-7′-glucoside, while derivatives of quercetin and kaempferol abounded in *D. tenuifolia*. Only two compounds were shared by both species, i.e., isorhamnetin-3,4-diglucoside and quercetin-3-glucoside.

**TABLE 2:** Content of phenolic compounds identified in leaves in leaves of rocket species (*Eruca sativa* and *Diplotaxis tenuifolia*) grown in soil and foliarly fertilized with selenate dosages ranging from 0 to 10 mg per plant.

Polyphenol (mg/100 g FW)	0	2.5	5	10
***Eruca sativa* Se treatment (mg per plant)**				
Q-3-glucoside	0.00 ± 0.00a	0.00 ± 0.00a	0.21 ± 0.04a	0.10 ± 0.01a
I-3-glucoside	0.11 ± 0.02b	0.23 ± 0.04a	0.07 ± 0.02b	0.08 ± 0.04b
K-3,4′-diglucoside	6.09 ± 0.98b	5.26 ± 0.91b	6.18 ± 0.44b	4.96 ± 0.25a
I-3,4′-diglucoside	0.49 ± 0.17b	0.53 ± 0.08b	0.41 ± 0.11b	0.44 ± 0.15a
K-3-(2-sinapoil-glucoside)-4′-glucoside	0.46 ± 0.05a	0.41 ± 0.12a	0.30 ± 0.02a	0.14 ± 0.04b
Q-3-glucoside 3′ (6-sinapoilglucoside)	0.00 ± 0.00a	0.09 ± 0.03a	0.24 ± 0.09b	0.09 ± 0.04b
K-3-sinapoyl sophoroside-7′-glucoside	0.15 ± 0.01b	0.38 ± 0.02a	0.38 ± 0.06a	0.21 ± 0.01a
K-3-sinapoil-triglucoside-7-glicoside	2.35 ± 0.17a	2.51 ± 0.19a	3.29 ± 0.27b	2.99 ± 0.16a
Total phenolic compounds	9.65 ± 0.98a	9.41 ± 0.77a	11.08 ± 0.55b	9.12 ± 0.46a
***Diplotaxis tenuifolia* Se treatment (mg per plant)**				
1-Sinapolyglucoside	0.00 ± 0.00a	0.00 ± 0.00a	0.44 ± 0.07a	0.62 ± 0.02a
Q-3-glucoside	0.12 ± 0.01a	0.18 ± 0.06a	0.07 ± 0.03a	0.08 ± 0.04a
Q-3,4′-diglucoside	0.13 ± 0.04b	0.39 ± 0.06a	0.16 ± 0.04b	0.12 ± 0.01b
I-3,4′-diglucoside	0.18 ± 0.02b	0.63 ± 0.09b	1.10 ± 0.20b	0.89 ± 0.18a
Q-3,3′,4′-triglucoside	0.23 ± 0.03b	0.19 ± 0.04b	0.13 ± 0.06b	0.23 ± 0.01a
Q-3,4′-diglucoside 3′ (6-sinapoil-glucoside)	0.00 ± 0.00a	0.00 ± 0.00a	0.19 ± 0.05a	0.11 ± 0.01b
Q-3-(2-ferulok glucoside)-3′-(6-sinapolyglucoside)-4′-glucoside	0.93 ± 0.33a	1.55 ± 0.16a	0.29 ± 0.17b	0.21 ± 0.17b
Total phenolic compounds	1.59 ± 0.36c	2.95 ± 0.23a	0.047 ± 0.22b	0.061 ± 0.22b

Data represent the mean of four biological replicates. Different letters along rows indicate significant differences (*p* < 0.05, ±STD) among treatments.

K, kaempferol; Q, quercetin; I, isorhamnetin.

In the analyzed samples, *E. sativa* contained more phenolic compounds than *D. tenuifolia.* However, while the total content of phenols was not substantially affected by Se fertilization in *E. sativa* plants, a substantial increase over control plants was evident in *D. tenuifolia* administered with Se, especially when supplied at the low dosage (2.5 mg per plant, +46%). These results mainly derive from the different behavior of individual phenol compounds in the two rocket species. In *E. sativa*, the content of kaempferol-3-sinapoyl diglucoside-7′-glucoside, quercetin-3-glucoside, kaempferol-3-snap sophoroside-7′-glucoside and quercetin-3-glucoside-3′-(6-sinapoyl-glucoside) slightly increased, while isorhamnetin-3-glucoside and kaempferol-3-(2-sinapoyl-glucoside)-4′-glucoside decreased. In *D. tenuifolia*, phenol compounds increased with Se application except for quercetin-3,3′,4′-triglucoside, which was almost unchanged, and quercetin-3-(2-feruloilglucoside)-3′-(6-sinapoil-glucoside)-4′-glucoside whose content conversely decreased.

### Content of selenium, nitrogen, carbon, and sulfur in soil after foliar selenium fertilization

The soil used to fill the pots initially contained a very low Se concentration (<0.5 mg kg^–1^) (Table 3). After the plant treatment with Se, we observed a weak increase in soil Se concentration, though it generally remained below 2.5 mg kg^–1^. The content of S in soil pots where *E. sativa* was cultivated decreased with increasing dosage of Se applied, and conversely increased in the soil where *D. tenuifolia* was grown. With respect to the content of N, inorganic C, and organic C, no significant differences were evident between pots containing plants fertilized with Se and pots with unfertilized plants.

**TABLE 3:** Content of total C, N, S and Se in soil where rocket species (*Eruca sativa* and *Diplotaxis tenuifolia*) were grown in soil and foliarly fertilized with selenate dosages ranging from 0 to 10 mg per plant.

Soil element	Time 0	0	2.5	5	10
***Eruca sativa* Se treatment (mg per plant)**					
C (%, w/w)	23.4 ± 0.10	21.88 ± 0.70a	20.85 ± 0.8a	20.76 ± 0.57a	21.51 ± 0.80a
N (%, w/w)	0.60 ± 0.08	0.57 ± 0.05b	0.50 ± 0.04a	0.48 ± 0.07b	0.52 ± 0.06b
S (%, w/w)	0.18 ± 0.01	0.16 ± 0.01b	0.16 ± 0.01b	0.14 ± 0.01ab	0.13 ± 0.00a
Se (mg kg^–1^)	0.02 ± 0.00	0.00 ± 0.00b	1.30 ± 0.36a	1.40 ± 0.20a	1.25 ± 0.13a
***Diplotaxis tenuifolia* Se treatment (mg per plant)**					
C (%, w/w)	23.4 ± 0.10	23.09 ± 0.00a	22.37 ± 0.10a	21.60 ± 0.17a	22.68 ± 0.10a
N (%, w/w)	0.60 ± 0.08	0.53 ± 0.01a	0.52 ± 0.06a	0.51 ± 0.03a	0.57 ± 0.04a
S (%, w/w)	0.18 ± 0.01	0.13 ± 0.01b	0.17 ± 0.01a	0.16 ± 0.01a	0.17 ± 0.02a
Se (mg kg^–1^)	0.02 ± 0.00	0.01 ± 0.00b	1.21 ± 0.30a	1.35 ± 0.27a	1.32 ± 0.35a

The initial concentration of C, N, S and Se is reported in brackets. The soil samples were analyzed in triplicates. Statistical differences are indicated by different letters (*p* < 0.05, ±STD). Comparison are made only between values measured at the end of the experiment.

## Discussion

In this study, we evaluated the effect of foliar application of selenate at different dosages on the accumulation of Se and on the content and profile of beneficial phytochemicals in two species of rocket. The need to study the content of different plant constituents after Se biofortification is to establish if the potential changes induced by the increased amount of Se in plants depending on the method of Se application could significantly impact other health-promoting nutritional components.

Using different methods of Se supplementation to the same plant species can result in distinct outcomes (Schiavon et al., 2016). In previous work, Se was applied to *E. sativa* and *D. tenuifolia* plants grown in hydroponics by adding selenate to the nutrient solution (Dall’Acqua et al., 2019). In that case, we found that such a method of Se administration was effective in enriching both species in Se, but elevated Se dosages (≥10 μM) determined a too high Se accumulation in leaf tissues resulting in plant material that cannot be considered completely safe. Furthermore, *D. tenuifolia* accumulated more Se than *E. sativa* likely because of greater S uptake that warranted a high abundance of S and S-containing compounds (Cys, GSH, and GLS) in plant tissues. In the present study, we did not observe differences in Se accumulation between *E. sativa* and *D. tenuifolia* when plants were foliarly supplied with low Se dosages (2.5 and 5 mg per plant), but at high Se supply (10 mg per plant) *E. sativa* revealed to accumulate significantly more Se. A possible explanation could lie in the fact that the leaf area of *E. sativa* plants is larger compared to *D. tenuifolia*, and thus would result in a greater number of transcuticular pores and stomata on the leaf surface that mediate the entry of Se into the mesophyll tissue (Reynoud et al., 2021).

Selenium was mainly determined in the leaf organs of both species, consistent with the method of Se supplementation, but was also partly accumulated in the root apparatus, indicating the capacity of rocket to efficiently translocate Se across the phloem. So far, little is known about the redistribution of selenate through the phloem (Trippe and Pilon-Smits, 2021), but the high-affinity sulfate transporter SULTR1;3 that localizes to phloem companion cells in both roots and shoot and is involved in the delivery of sulfate from source to sink organs (Yoshimoto et al., 2002) might have a role in Se mobilization, as its expression was recently reported to be upregulated in response to selenate treatment in wheat (Boldrin et al., 2016). Se transportation from leaves to roots was previously reported in other plant species like radish (Schiavon et al., 2016) and carrot (Kápolna et al., 2009). We exclude that Se in roots derived from Se deposition in soil after selenate treatment, as no significant variation in the natural content of Se in soil was determined. It is likely that conveying Se to the roots could be a strategy of rocket plants to limit Se accumulation in the leaves, thus reducing the toxicity of excess Se in photosynthetic tissues. Indeed, many plants tend to accumulate metals and metalloids in the roots to prevent their toxicity and ROS overgeneration in the shoot (Dal Corso et al., 2013).

The low Se dosage (2.5 mg per plant) had positive effects on both the leaf and root biomass of the two rocket species. Such a beneficial effect of Se on plant growth at low concentration is well-known and thought to be associated with cell membrane development, stimulation of photosynthetic efficiency in terms of faster electron transport rate along photosystems and chloroplast development (Schiavon and Pilon-Smits, 2017), and upregulation of antioxidant metabolism (Chauhan et al., 2019). It is noteworthy that the growth of rocket plants was more pronounced when they were cultivated in soil than in the hydroponic setup. Several possible reasons may have caused this difference. First, the plants grown in soil were 1 week older than plants placed in hydroponics; second, the plants raised in hydroponics were transplanted from the agar medium, which may have generated temporary, albeit mild, stress; third, plants within the same pot in hydroponics could have competed for nutrient resources limiting growth; fourth, certain soil rhizosphere microorganisms may have stimulated plant growth and/or contributed to alleviate Se stress (de Souza et al., 1999; White and Broadley, 2009; El Mehdawi and Pilon-Smits, 2012; Winkel et al., 2015; Ye et al., 2020).

The application of the low selenate dosage (2.5 mg Se per plant) through foliar spray also appeared to be a worthy approach to enrich *E. sativa* and *D. tenuifolia* with Se, but still provided a too high amount of this element to consumers. Indeed, the consumption of about only 4–5 g of leaf fresh material derived from *E. sativa* or *D. tenuifolia* plants would meet the daily consumers’ requirement for Se, which ranges from 55 to 70 μg. On the other hand, the supply of higher Se dosages (5 or 10 mg Se per plant) to rocket species caused a very high Se accumulation in the shoot, and therefore only little amounts of leaf material from *E. sativa* (<1–1.93 g leaf FW) or *D. tenuifolia* (1.47–2.19 g leaf FW) could be safely consumed. In any case, as the amount of leaf material that is recommended is overall very small, the fresh leaves of either species could be more suitably added to mixed salads for consumption. These results differ compared to those obtained in the hydroponic study, where *E. sativa* accumulated much less Se in leaves, and thus greater consumption of leaf fresh material from this species could be recommended (Dall’Acqua et al., 2019). Perhaps, the accumulation of Se in leaves of *E. sativa* growing in hydroponics was constrained by the Se uptake capacity of the root system and the further Se root to shoot translocation rate. Conversely, in the current study, the direct application of selenate on the leaf surface ensured a faster Se absorption by this species, even though a little part of Se could also have been lost by leaf washing after harvest.

Selenocysteine (SeCys) was the unique Se amino acid determined in both rocket species, as in the hydroponic study. However, the amount of this compound was substantially higher when plants were foliarly fertilized with Se, and values were comparable between *E. sativa* and *D. tenuifolia*. This means that foliar Se fertilization could be more efficient in enriching rocket in this form of organic Se. This is important to note, because organic Se species, such as Se-amino acids, are considered the most efficient form of biofortification (Davis, 2012). The reduction of free SeCys may be due to its increased incorporation into proteins in place of Cys, which apparently was rather used more for the steady synthesis of GSH.

The impact of Se biofortification on the content of S and S-containing compounds (Cys and total GLS) was the opposite in the two rocket species. Similarly to findings obtained in the hydroponic study, Se and Cys were more abundant in *D. tenuifolia* than *E. sativa* under no Se or low Se treatment, and *E. sativa* plants supplied with the high Se dosage exhibited the elevated capacity to re-mobilize S, which was early found to be dependent on the up-regulation of the low affinity sulfate transporter SULTR2;1, involved in Se/S root to shoot translocation (Dall’Acqua et al., 2019). However, here we found the accumulation of Cys increased at any Se dosage applied, thus suggesting that enhanced S assimilation can be induced by foliar selenate treatment. It cannot be ruled out that part of Cys may also derive from the turnover of proteins that removes those abnormal or misfolded (Vierstra, 1996). The enhanced accumulation of Cys in *E. sativa* probably served to prevent a decline in the amount of other essential S-compounds such as Met and GSH, which require Cys as a precursor for their synthesis. Consequently, Met-derived GLS were unaffected by Se treatment. These results are different from those reported in the same species supplemented with Se in hydroponics (Dall’Acqua et al., 2019), where selenate treatment affected the expression of genes involved in the S assimilation pathway, consequently reducing the synthesis of Cys, GSH, Met, and GLS, and indicates that the foliar or soil supplementation of Se can yield to different plant responses.

In *D. tenuifolia*, the Cys content followed an opposite trend compared to *E. sativa* as it was reduced by the high selenate treatment, while S was decreased at any applied selenate dosage. The decrease in S can be due to an effect of foliar Se treatment on S acquisition by roots, as the amount of S in the soil of plants treated with Se was higher than the soil used to grow untreated plants. This result indicates the existence of a long-distance effect of Se applied on leaves on the Se/S root uptake transport systems in this species. Although S decreased in roots, plants treated with 2.5 or 5 mg Se per plant maintained a steady level of S in leaves, ensuring a constant synthesis of Cys. The decrease in Cys content that occurred with high Se treatment was in line with the reduction of S in leaves, but the fact that the GSH content was unchanged suggests that Cys was the preferential precursor for GSH over other compounds. In support of this, the content of Met decreased at the high selenate dosage in *D. tenuifolia*, consistent with the reduction of GLS, most of which were Met-derived.

The reduction of GLS content is a disadvantage of Se fertilization and could have potential ecological implications. GLS are involved in the defense of plants against herbivores and pathogens and their decrease could limit the plant’s capacity to prevent attacks by these organisms, with consequent yield losses. To some extent, the accumulated Se may take over the protective role: even levels as low as 1 mg kg^–1^ dry weight (DW) have already been shown to be protective against generalist herbivores, due to deterrence and toxicity (Hanson et al., 2004). On the other side, GLS are also responsible for the typical bitter taste of rocket. So, Se may reduce the bitterness of wild rocket making it potentially attractive for some consumers.

The Se foliar treatment altered the content and profile of free amino acids and phenolic compounds in the two rockets species, with some differences. Early studies reported changes in the content of such compounds due to Se (Djanaguiraman et al., 2005; Schiavon et al., 2016; Dall’Acqua et al., 2019; Yin et al., 2019). In the case of free amino acids, the content of most of them was increased by one or more Se dosages applied. Phenylalanine, in particular, was increased in both rocket species treated with high Se dosage; this amino acid is a substrate for aromatic GLS, but we did not identify any GLS derived from phenylalanine, though their existence in rocket is documented (Bell and Wagstaff, 2014; Bell et al., 2015; Toledo-Martín et al., 2017). Possibly, phenylalanine was preferentially used for the synthesis of phenols, as the enzyme phenylalanine ammonia-lyase (PAL) that uses this amino acid as a substrate can be induced by Se (Astaneh et al., 2018). A special note should be made for proline, because in the previous hydroponic study its content was substantially increased by Se in *D. tenuifolia*, while decreased in *E. sativa*. In the present study, however, we obtained the opposite result. Because proline acts as an osmolyte in cells to counteract osmotic stress, it is possible that its increase in *E. sativa* contributed along with other major osmolytes (e.g., non-structural sugars) to the plant’s need to alleviate the osmotic imbalance due to the higher accumulation of Se in its tissues. In addition, proline exerts a protective effect on phospholipids, plasmalemma, mitochondria and plastid membranes (Naliwajski and Skłodowska, 2021), and it can contribute to the scavenging of hydroxyl radicals via a proline cycle (Signorelli et al., 2014). This cycle could also be coupled to the pentose phosphate pathway that generates erithrose-4-phosphate, a precursor of the shikimate pathway that leads to the production of chorismate, which is the branch point between primary and secondary metabolism and can promote the synthesis of secondary metabolites, including phenols (Shetty and Wahlqvist, 2004). Previously, a significantly more negative osmotic potential was found when *Brassica juncea* plants were treated with Se (unpublished data).

With respect to the phenolic compounds, various studies report contrasting results concerning the effect of Se on their synthesis and accumulation, which can depend on the form of Se applied to plants, the plant species and/or the method of Se administration (Robbins et al., 2005; Tian et al., 2016; D’Amato et al., 2018, 2020; Dall’Acqua et al., 2019). In our study, the effect of Se foliar administration on phenolic compounds had different effects in the two rocket species. Similar to the early hydroponic study, the net content of total phenolics in *E. sativa* was almost unchanged, although the level of some individual compounds increased and others decreased. In *D. tenuifolia*, the content of most phenolic compounds tested increased with Se supplementation, especially when plants were treated with the low Se dosage. This trend is opposite compared to those described for plants supplied with Se in hydroponics, as observed in the case of Cys, GLS, and certain free amino acids.

## Conclusion

In conclusion, this study highlights the relevance of the method of Se supplementation in shaping the responses of the same plant species in terms of Se enrichment, S assimilation, synthesis, and accumulation of primary (e.g., amino acids, GSH) and secondary metabolites (GLS, phenolic compounds). Our results indicate that a single method of Se administration may not be the most suitable for a given plant species because the responses to different Se treatments could be very different, sometimes opposite. Therefore, before starting a large-scale biofortification program it would be more appropriate to carry out preliminary small-scale trials using the species to be enriched with Se to identify the most suitable method for applying Se. This must be done with the aim to biofortify plants with Se without compromising the content of other nutritionally valuable phytochemicals in the edible products.

## Data availability statement

The raw data supporting the conclusions of this article will be made available by the authors, without undue reservation.

## Author contributions

MS: investigation, methodology, conceptualization, data curation, writing original draft, review and editing, and funding acquisition. EP-S: investigation, methodology, and review and editing. SN: review and editing and funding acquisition. SD: methodology, data curation, and review and editing. All authors contributed to the article and approved the submitted version.
