# Supplementary material for: Foliar selenium fertilization alters the content of dietary phytochemicals in two rocket species
Source: Front Plant Sci. 2022 Aug 31;13:987935. doi: 10.3389/fpls.2022.987935 (PMC9470978; doi:10.3389/fpls.2022.987935)
Supplement: Supplementary file 1 [file Data_Sheet_1.PDF]

Supplementary Material

Table 1S. Fragmentation pattern of glucosinolates identified in leaves of *E. sativa* and *D. tenuifolia plants*. Compounds were detected in positive ion mode observing sodium adducts $[M+Na]^+$ or $[2M+Na]^+$, or potassium adduct $[M+K]^+$ or hydrogen adduct $[M+H]^+$ pseudomolecular ions. Abbreviation DBM-GLS indicate Dimeric-4-mercaptobutyl glucosinolate.

Glucosinolate	Fragmentation	$[M+Na]^+$	$[M+H]^+$	$[M+K]^+$	$[2M+Na]^+$
**Glucoraphanin**	316,218,136	380			
**Glucoalissin**		394			
**Glucoerucin**	202	364		380	
**Glucosativin**			328		
**Neoglucobrassicin**		421			
**Hydrossyglucobrassicin**					791
**Methoxyglucobrassicin**			399	437	
**DMB-GLS**	513, 479	675		691	

Table 2S. Fragmentation pattern of phenolic compounds identified in leaves and roots of rocket plants. [M+H]$^+$ - protonated adduct pseudomolecular ions. K= kaempferol; Q =quercetin.

Polyphenol	Fragmentation	[M+H]$^+$
**K-3-sinapoil-triglucoside-7-glicoside**	301, 179	1139
**Q-3-glucoside**	976, 815, 609, 447	463
**Q-3,4’-diglucoside**	285, 257, 151	625
**K-3,4’-diglucoside**	447, 285, 255, 151	609
**I-3,4’-diglucoside**	447, 315, 300, 285	639
**K-3-sinapoylsophroside-7’-glucoside**	284, 429, 489, 609	977
**K-3-glucoside**	785, 285, 591	447
**I-3-glucoside**	314, 285, 271	477
**Q-3-glucoside 3’ (6-sinapoilglucoside)**	609, 447	831
**K-3-(2-sinapoil-glucoside)-4’-glucoside**	669, 463, 301	815
**1-sinapolyglucoside**	223, 247	385
**Q-3,3’,4’-triglucoside**	625, 463	787
**Q-3,4’-diglucoside 3’ (6-sinapoil-glucoside)**	831, 669, 463	993
**Q-3-(2-feruloilglucoside)-3'-(6-sinapolyglucoside)-4'-glucoside**	301, 463, 669, 1037	1169
